# Oral Microbiota Distinguishes Acute Lymphoblastic Leukemia Pediatric Hosts from Healthy Populations

**DOI:** 10.1371/journal.pone.0102116

**Published:** 2014-07-15

**Authors:** Yan Wang, Jing Xue, Xuedong Zhou, Meng You, Qin Du, Xue Yang, Jingzhi He, Jing Zou, Lei Cheng, Mingyun Li, Yuqing Li, Yiping Zhu, Jiyao Li, Wenyuan Shi, Xin Xu

**Affiliations:** 1 State Key Laboratory of Oral Diseases, West China Hospital of Stomatology, Sichuan University, Chengdu, China; 2 Department of Pediatric Hematology and Oncology, West China Second University Hospital, Sichuan University, Chengdu, China; 3 Department of Pediatric Dentistry, West China Hospital of Stomatology, Sichuan University, Chengdu, China; 4 UCLA School of Dentistry, Los Angeles, California, United States of America; Instutite of Agrochemistry and Food Technology, Spain

## Abstract

In leukemia, oral manifestations indicate aberrations in oral microbiota. Microbiota structure is determined by both host and environmental factors. In human hosts, how health status shapes the composition of oral microbiota is largely unknown. Taking advantage of advances in high-throughput sequencing, we compared the composition of supragingival plaque microbiota of acute lymphoblastic leukemia (ALL) pediatric patients with healthy controls. The oral microbiota of leukemia patients had lower richness and less diversity compared to healthy controls. Microbial samples clustered into two major groups, one of ALL patients and another of healthy children, with different structure and composition. Abundance changes of certain taxa including the Phylum *Firmicutes*, the Class *Bacilli*, the Order *Lactobacillales*, the Family *Aerococcaceae* and *Carnobacteriaceae*, as well as the Genus *Abiotrophia* and *Granulicatella* were associated with leukemia status. ALL patients demonstrated a structural imbalance of the oral microbiota, characterized by reduced diversity and abundance alterations, possibly involved in systemic infections, indicating the importance of immune status in shaping the structure of oral microbiota.

## Introduction

Leukemia is a cancer of the early blood-forming cells. Acute lymphoblastic leukemia (ALL), a malignant disorder of lymphoid progenitor cells, is the most common type of leukemia among children, accounting for 75% of all childhood leukemia and 25% of all malignancy in childhood [1]. Among some individuals leukemia first manifests in oral cavity [2], [3]. Oral manifestations that frequently occur in leukemia patients include gingival bleeding, oral ulceration, gingival enlargement, candidiasis and periodontitis [4], [5], [6]. Oral microbes are believed to be involved in the occurrence or exacerbation of such complications [7]. Certain oral microbiota have been shown to contribute to septicemia, which might delay antineoplastic treatment, compromise treatment efficiency, or even jeopardize the patients' life [8], [9]. Therefore, an adequate treatment of oral lesions could lead to a more favorable resolution of both oral and systemic diseases. At present, limited knowledge is available on the oral microbiota of leukemia patients. Previous studies dependent on a culture-based approach have mainly focused on limited cultivable bacterial species and failed to determine the holistic pattern of oral microbiota in leukemia patients [10], [11], [12], [13]. Furthermore, because most previous studies were primarily focused on the effect of antineoplastic treatment on oral microbes, limited information can be obtained on the oral microbiota *per se* under the diseased condition. To determine the role of oral cavity microbiota to local and systemic complications in ALL patients, it is first necessary to generate a more complete picture of the oral cavity microbiota population that is specific to this disease.

In ALL patients, lymphoid progenitor cells are affected, which can partially impair the immune system of the host [14], [15]. It is known that microbiota structure within a host is determined by both host and environmental factors. If any one of these factors is greatly perturbed, a drastic composition shift is expected in the oral microbiota, and disease occurs. In the human host, the role of health status in shaping the composition of oral microbiota is still largely unknown. Characterization of the oral microbiome in ALL patients provides information about oral microbiota-host interactions in immunocompromised individuals, and thus contributes to better management of oral and systemic complications associated with immunodeficiency.

To elucidate how a compromised host immune status leads to a perturbed microbial homeostasis necessitates a thorough comparison of the oral microbial composition of ALL patients and ALL-free population. Remarkable advances in high-throughput sequencing have recently improved practicality in analysis of microbiota from a variety of biological and environmental samples under various conditions. Whereas conventional culture-dependent approaches underestimate microbial composition, new culture-independent molecular techniques are capable of investigating entire bacterial communities and characterizing the biodiversity of oral microbiota. High-throughput sequencing has been widely and successfully applied to the exploration of oral microbial diversity in health [16], [17], [18] and disease-associated communities from those associated with dental caries [19], [20], periradicular lesions [21], atherosclerosis [22], and head-neck tumor undergone radiotherapy [23], [24]. However, application of these techniques in investigating the oral microbiome under immunocompromised conditions has so far been limited. In this study, we characterized the biodiversity of supragingival plaque microbiota in pediatric clinic ALL patients using a high-throughput sequencing technique (454 pyrosequencing). The oral microbiota compositional profiles were compared to those of ALL-free healthy subjects.

## Methods

### Ethics Statement

This study was approved by the Ethics Committee of State Key Laboratory of Oral Diseases, Sichuan University, Chengdu, China, and conducted in compliance with the Declaration of Helsinki. Written informed consent was obtained from the parents or guardians of all subjects before the study.

### Study Population

Potential study subjects of the leukemia group were selected from a group of patients who were newly diagnosed with acute lymphoblastic leukemia (ALL) in the Department of Pediatric Hematology and Oncology, West China Second University Hospital, Sichuan University. All subjects received no previous antineoplastic treatment. Demographic information was obtained and oral examination was performed. A gender-, age-, and caries status matched healthy counterpart for each leukemia child was recruited from Department of Pediatric Dentistry, West China Hospital of Stomatology, Sichuan University. The detailed eligible criteria are presented in Table 1. Detailed clinical data of each subject is in Table S1.

**Table 1:** Admission criteria.

ALL patients group	
Inclusion criteria	Exclusion criteria
(1) Newly diagnosed with acute lymphoblastic leukemia based on bone marrow samples	(1) Previous antineoplastic treatment
(2) No more than eighteen years old	(2) Receiving antibiotics within 3 months before the study
(3) Free of other systemic diseases (including systemic infection)	(3) Local antimicrobial treatment within 2 weeks
(4) Written informed consent	

Healthy control group	
Inclusion criteria	Exclusion criteria
(1) Age, socioeconomic and caries status comparable with ALL patients	(1) Systemic or local disorders that cause oral mucosal lesions such as lichen planus
(2) Free of systemic diseases	(2) Periodontal pockets equal to or greater than 4 mm
(3) Written informed consent	(3) Acute oral infection such as dental abscess at enrollment
	(4) Evidence of oral candidiasis
	(5) Receiving antibiotics within 3 months before the study
	(6) Local antimicrobial treatment within 2 weeks

ALL patients: acute lymphoblastic leukemia patients.

### Sample Collection and Oral Examination

For each of the 26 subjects, microbial samples were collected at the same time of day, approximately 2 hours after breakfast, using the method mentioned in the Manual of Procedures for Human Microbiome Project (http://hmpdacc.org/tools_protocols/tools_protocols.php) with minor modifications. Briefly, the sampling sites, the teeth in upper right and lower left quadrants or upper left and lower right quadrants, were isolated with cotton rolls and dried before sampling. A sterile Gracey curette was used to collect a pooled supragingival plaque sample from the mesial surfaces of each of these teeth in turn. The collected plaque samples were released from the curette by agitation in 700 µl of TE buffer (10 mM Tris-Cl [pH 7.5] and 1 mM EDTA). The microbial samples were immediately transported on ice to the laboratory and stored at −80°C until further DNA extraction and pyrosequencing analysis.

Oral conditions of each subject, including dentition status, number of teeth, plaque index, caries status, and presence or absence of gingivitis/periodontitis, were recorded after sampling. The presence of caries was further confirmed by periapical radiographs. Plaque-Check+pH kit (GC Corporation, Japan) was used for testing plaque fermentation based on a colourimetric readout of the pH of supragingival plaque corresponding to each sampling site [25].

### DNA Extraction

Bacteria were pelleted from dental plaque samples by centrifugation (Thermo Electron Corporation, Boston, MA, USA) at full speed (more than 10,000×g) for 10 min. Bacterial DNA was extracted using QIAamp DNA Micro Kit (QIAGEN, Hilden, Germany) according to the manufacturer's instructions with minor modification. Briefly, 30 µl lysozome solution (50 mg/ml) was added to the mixture at the first step to increase the yield of bacterial DNA from Gram-positive bacteria by hydrolyzing the peptidoglycan cell wall. The amount of DNA extracted per sample was determined using Quant-iT PicoGreen dsDNA Assay Kit (Invitrogen, Carlsbad, USA). Size and integrity of DNA were checked by 1% (w/v) agarose gel electrophoresis in 0.05‰ (v/v) GoldView. The extracted DNA was then stored at −20°C before further analyses.

### Pyrosequencing and Data Analysis

The 16S rRNA hypervariable V1–V3 region was amplified using polymerase chain reaction (PCR) with the forward primer 8F (59-AGAGTTTGATCCTGGCTCAG-39) and reverse primer 533R (59-TTACCGCGGCTGCTGGCAC-39) [18]. Unique 10-bp barcodes were incorporated into the reverse primers so that sequences of different samples can be differentiated. Each 25 µl PCR reaction consisted of 1 µl of forward primer (10 µmol/l), 1 µl of barcoded reverse primer (10 µmol/l), 0.5 µl of dNTP mix (10 mmol/l), 2.5 µl of FastStart 10× buffer with 18 mmol/l of MgCl_2_, 0.25 µl of FastStart HiFi Polymerase (5 U/µl), 1 µl of genomic DNA, and 18.75 µl of water. FastStart HiFi Polymerase, FastStart 10× buffer with MgCl_2_ and dNTP mix were included in the FastStart High Fidelity PCR System, dNTP Pack (Roche Applied Science). The PCR amplification was performed using the following program: 3 minutes of initial denaturation at 94°C, followed by 25 cycles of denaturation (94°C for 15 seconds), annealing (56°C for 30 seconds) and extension (72°C for 30 seconds) with a final extension of 5 minutes at 72°C. To obtain sufficient PCR products for pyrosequencing, the amplicons of three replicates were pooled. After PCR, amplicons were gel purified using MinElute Gel Extraction Kit (QIAGEN), and then quantified with Quant-iT PicoGreen dsDNA Assay Kit (Invitrogen).The PCR products were combined in equimolar ratios to create a DNA pool used for pyrosequencing. Pyrosequencing was performed according to standard Roche 454 GS-FLX protocols [26].

To optimize raw sequences, the sequence analyzing programs Seqcln (http://sourceforge.net/projects/seqclean/) and MOTHUR (version 1.30.0; http://www.mothur.org) were applied to the data. The primer sequences and 10-bp barcode were removed. The sequences were checked and low quality sequences (quality score <25) were discarded. Sequences that contained ambiguous bases, incorrect primer sequence, or identified to be shorter than 200 bp, or homologous sequences longer than six nucleotides were also removed. UCHIME (http://drive5.com/uchime) was used to detect potentially chimeric artifacts. Sequences were clustered to operational taxonomical units (OTU) using CD-HIT-EST at 97% similarity level. RDP NaÏve Bayesian Classifier was applied to perform read level taxonomic assignments with an 80% bootstrap score [27]. Qualified sequences were submitted to the SILVA database (SILVA 111; http://www.arbsilva.de) for taxonomic alignment. Community richness (ACE, Chao1) and diversity indices (Simpson diversity index) were determined by the MOTHUR program at 97% similarity level. The statistical significance of these indices was determined by SPSS 19.0 (SPSS Inc, Chicago, IL, USA) with nonparametric Mann-Whitney U test for independent samples. The heatmap profile was generated by the R program (http://www.r-project.org/). For phylogeny-based cluster comparisons, principal coordinate analysis (PCoA) plots were generated with the distance matrix calculated using the weighted UniFrac algorithm. The composition of the microbial communities present in the samples from the ALL patients (n = 13) and control (n = 12) groups were analyzed using unweighted and weighted UniFrac analysis [28], [29] and Parsimony p-tests [30]. Metastats was used to compare the relative abundance of each taxon at different taxonomic levels between ALL patients and healthy children [31], with the *p* value threshold set at 0.05 and the q value threshold at 0.5 [32].

### Sequence Deposition

Sequences were deposited in the NCBI sequence read archive (http://www.ncbi.nlm.nih.gov/Traces/sra/) under accession number SRP034724.

## Results

### Richness and Diversity of Oral Microbiota in ALL patients and Healthy Children

In total, 356000 qualified sequence reads were obtained and used for analysis, with an average of 13692 sequence reads for each sample. One healthy subject sample (H04) had low sequence counts making the depth of pyrosequencing not comparable to other samples. Thus to avoid potential bias caused by uneven sequence depth, this sample was not subjected to further bioinformatics analyses, leaving 13 ALL patients and 12 healthy control subjects samples for downstream data analyses.

The species-level operational taxonomic units (OTUs) and richness and diversity estimators were generated for each sample (Table S2). Clustering the unique sequences into OTUs at a 0.03 dissimilarity level formed 2280 OTUs per microbiome on average. Richness and diversity estimation of 16S rRNA gene libraries at 97% similarity was calculated and compared between two groups (Table 2). Comparison of species-level OTUs between ALL patients and healthy children revealed that the ALL patients group exhibited lower richness (*p* = 0.004). Furthermore, community richness estimators (Chao 1 and ACE) also revealed a statistically significant lower estimate of richness for ALL patients compared with healthy children. ALL patients showed less diversity compared with healthy subjects as demonstrated by comparing the Simpson Index between the oral microbiota of the two groups (*p* = 0.019).

**Table 2:** Comparison of richness and diversity estimates of 16S rRNA gene libraries at 97% similarity between the acute lymphoblastic leukemia patients and healthy subjects.

Variables	ALL patients (n = 13)	Healthy control (n = 12)	*p* value*
OTUs	1778±874	2824±647	0.004
Chao 1^a^	4738.27±2049.02	7215.11±1505.13	0.003
ACE^b^	8305.50±3336.40	12455.82±2400.76	0.004
Simpson^c^	0.0393±0.0246	0.0168±0.0082	0.019

Applicable values are means±SD. ALL patients: acute lymphoblastic leukemia patients.

a,b Richness estimators (Chao 1 and ACE) were calculated using MOTHUR.

c A higher number indicates less diversity.

*Independent sample nonparametric Mann-Whitney U test was used.

### Structural Comparisons of Oral Microbiota between ALL Patients and Healthy Subjects

**Figure 1:**
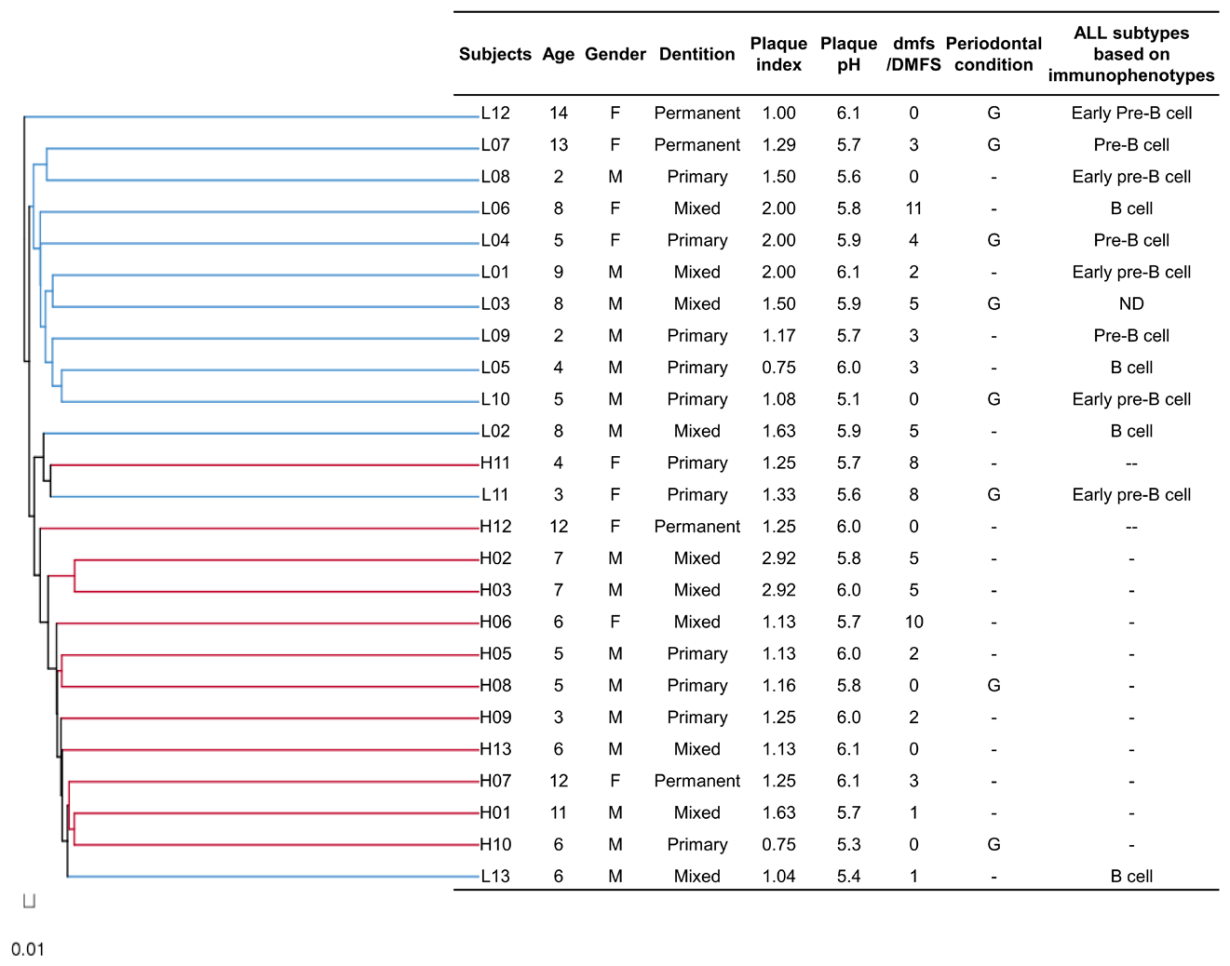
Dendrogram of OTU composition obtained from acute lymphoblastic leukemia-affected children and healthy controls. The dendrogram (left section) indicates the similarity of the microbial communities of supragingival dental plaque between subjects according to their OTU composition, as determined using the Jaccard index (MOTHUR). A summary of patients' clinical information is presented in the right section. H, healthy children. L, acute lymphoblastic leukemia-affected children. dmfs/DMFS, decayed-missing-filled surfaces index in primary and permanent teeth, respectively. G, gingivitis. ND, no data obtained.

**Figure 2:**
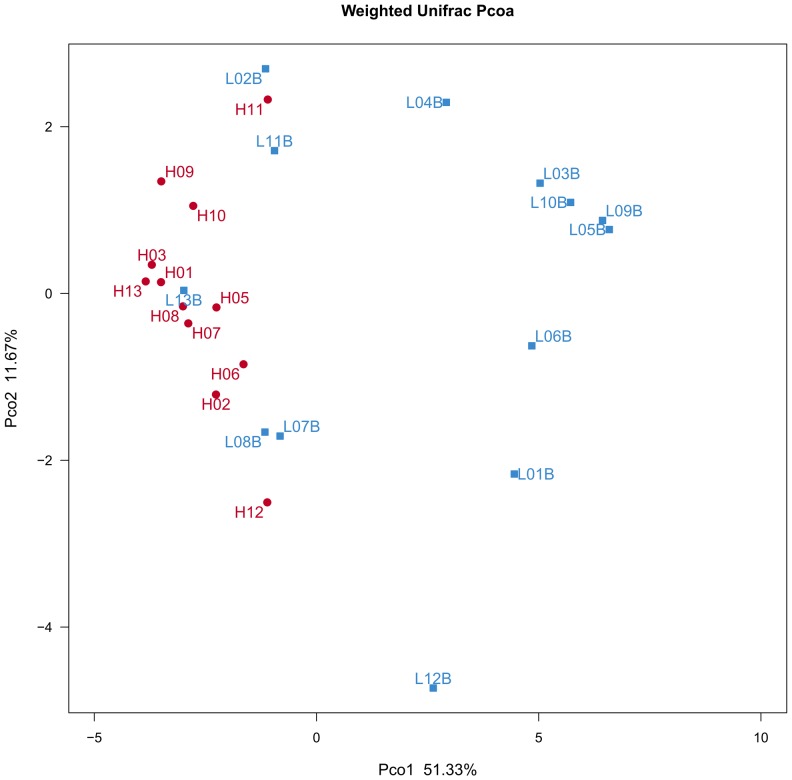
Weighted Unifrac PCoA analysis. The first two principal coordinates (PCo1 and PCo2) from the principal coordinate analysis of weighted UniFrac are plotted for each sample. The variance explained by the PCos is indicated in parentheses on the axes. H, healthy children. L, acute lymphoblastic leukemia-affected children.

No statistically significant difference regarding the demographic information and oral health conditions was observed between the ALL group and the healthy control (*p*>0.05), albeit the ALL patients have a higher incidence of periodontitis compared with the healthy subjects (Table 3). We compared the oral microbial communities present within each of the 13 ALL patients and 12 control subjects using three different types of cluster analysis: OTU-based, taxonomy-based (at the order level) and phylogeny-based cluster analyses. The dendrogram from the OTU-composition presented two clusters, with one cluster composed of leukemia oral samples and the other mainly comprised of healthy control samples (Figure 1). Moreover, the supragingival plaque samples from the ALL patients and healthy controls could be divided into two subsets based on microbiota composition and microbe abundance at the order level. This taxonomy-based clustering at the order level was better associated with the health or disease status (ALL-affected *vs.* ALL-free) of subjects as shown in Figure S1. Principal coordinate analysis (PCoA) based on the weighted UniFrac metric was also performed. Although 5 subjects from ALL patients group clustered with the healthy controls, a segregation trend for ALL patients and healthy subjects was observed, especially by principal coordinate P1 (Figure 2). To further validate the statistical significance of the aforementioned sample clustering, we performed three different types of pair-wise comparisons, including parismony test, Unifrac unweighted and Unifrac weighted analyses. A single NJ phylogenetic tree containing all 16S rRNA sequences from the samples from the 13 ALL patients and 12 control subjects (identical to that used for the PCoA analysis) was used as the input for all three methods. All three statistical approaches showed significant difference (parismony test, *p* = 0.006; Unifrac unweighted, *p* = 0.005; Unifrac weighted, *p*<0.001), indicating that the oral microbial structure of the ALL-affected population is distinct from that of the healthy controls.

**Table 3:** Demographic and oral health information of acute lymphoblastic leukemia and healthy subjects.

Variables	Characteristics	ALL patients (n = 13)	Healthy control (n = 12)	*p* value
Age (years)	Mean ± SD	6.69±3.82	7.00±3.05	0.806^a^
Dentition stage	Primary	6	5	0.975^b^
	Mixed	5	5	
	Permanent	2	2	
Sex	Male	8	8	1.000^c^
	Female	5	4	
Caries status	dmfs/DMFS	3.46±3.26	3.00±3.36	0.600^a^
Periodontal condition	Healthy	7	10	0.202^c^
	Gingivitis	6	2	
	Periodontitis	0	0	
Plaque index	Silness-Löe Index	1.41±0.41	1.48±0.70	0.743^a^
Plaque pH	Mean ± SD	5.75±0.28	5.85±0.23	0.350^a^

ALL patients: acute lymphoblastic leukemia patients. dmfs/DMFS: decayed-missing-filled surfaces index in primary and permanent teeth, respectively.

a Independent sample nonparametric Mann-Whitney U test was used.

b Pearson Chi-Square test was used.

c Fisher's Exact test was used.

### Taxonomy-Based Comparisons of Oral Microbiota between the Two Host Populations

**Figure 3:**
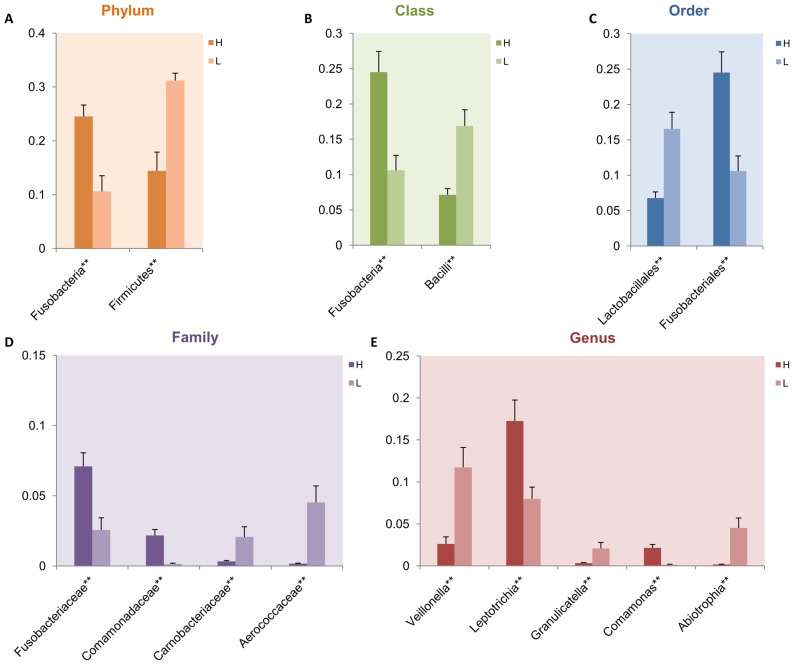
Differential relative abundance of bacterial taxonomy profiles of acute lymphoblastic leukemia and healthy subjects based on Metastats analysis. Comparisons were performed at each of the taxonomical levels of Phylum (A), Class (B), Order (C), Family (D) and Genus (E). Means of the relative abundance for each taxon at each taxonomical level between the healthy and acute lymphoblastic leukemia host-populations are compared, with a *p* value threshold set at 0.05 (*p<0.05; **p<0.01) and a q value threshold at 0.5. The discriminating taxa (*p*<0.05 and q<0.5) with relative abundance >1% for at least one group at each level are shown. H, healthy children. L, acute lymphoblastic leukemia-affected children.

Comparisons of oral microbiota between ALL patients and healthy subjects at each of taxonomical levels of phylum, class, order, family and genus were performed based on Metastats analysis. From the phylum level down to the Genus level, there were no ‘leukemia-specific’ or ‘healthy-specific’ taxa unique to either leukemia or healthy hosts. However, ‘leukemia-associated’ taxa (present in healthy and leukemia populations but differentially distributed) were detected, which were either ‘leukemia-enriched’ or ‘leukemia-depleted’ for certain microbe classes (Figure 3; Table S3). A total of 12 phyla were identified in oral microbiota of ALL patients and healthy children in our dataset, which were dominated by six major phyla, including *Proteobacteria*, *Firmicutes*, *Fusobacteria*, *Actinobacteria*, *Bacterioidetes* and candidate division TM7 (Figure S2). Particularly, notable differences in abundance between ALL patients and healthy subjects were found for the two phyla, i.e. *Firmicutes* (*p* = 0.001) and *Fusobacteria* (*p* = 0.003). *Firmicutes* was more abundant while *Fusobacteria* was less abundant in ALL patients compared to healthy children (Figure 3). Of all genera detected from oral cavity samples, *Abiotrophia* (*p* = 0.001), *Comamonas* (*p* = 0.001), *Granulicatella* (*p* = 0.002), *Leptotrichia* (*p* = 0.001) and *Veillonella* (*p* = 0.001) were significantly different between ALL patients and healthy subjects. Leukemia-enriched genera included *Abiotrophia*, *Granulicatella* and *Veillonella*, while *Comamonas* and *Leptotrichia* constituted leukemia-depleted genera (Figure 3).

Taking taxonomic lineages into account, two lineages were found to be more abundant in the ALL patients compared to the healthy children from the phylum down to the genus level (Table S3). The two leukemia-enriched taxonomic lineages included *Firmicutes* (*p* = 0.001) at Phylum, *Bacilli* (*p* = 0.001) at Class, *Lactobacillales* (*p* = 0.001) at Order, *Aerococcaceae* (*p* = 0.001) at Family and *Abiotrophia* (*p* = 0.012) at genus, as well as *Firmicutes* (*p* = 0.001) at Phylum, *Bacilli* (*p* = 0.001) at Class, *Lactobacillales* (*p* = 0.001) at Order, *Carnobacteriaceae* (*p* = 0.002) at Family and *Granullicatella* (*p* = 0.007) at Genus. Moreover, one leukemia-depleted lineage was found, consisting of *Fusobacteria* (*p* = 0.003) at Phylum, *Fusobacteria* (*p* = 0.001) at Class, *Fusobacteriales* (*p* = 0.001) at Order and *Fusobacteriaceae* (*p* = 0.002) at Family, but not *Fusobacterium* (*p* = 0.009; q>0.5) at Genus.

## Discussion

This study compared the oral microbial composition of acute lymphoblastic leukemia (ALL) and healthy hosts, contributing to a better understanding of the oral microbial profiles in immunocompromised patients. While there have been several reports investigating oral microbiota in leukemia patients [10], [11], [12], [13], [33], most of these studies relied on traditional culturing methods and were focused on only a few kinds of common bacteria, failing to characterize the overall pattern of oral microbiota in leukemia patients. In addition, most of these cross-sectional investigations were focused on the effect of antineoplastic treatment on oral microbiota, and limited information can be obtained regarding the oral microbiota under ALL-associated immunocompromised conditions. These limitations have severely confounded efforts to pinpoint leukemia-associated oral microbiota in patients.

Since gingivitis/periodontitis are common oral pathologies experienced by leukemia patients [4], [6], it is critical to distinguish whether a detected microbial change is driven by the global immune status or by the unavoidable existing periodontal pathology. We believe that compared to subgingival and salivary microbiota, which alter under periodontal pathologies [34], [35], the supragingival microbiota is less affected by gingivitis/periodontitis, but more by the host immune status given that the caries status is normalized. Therefore, in the present study, we sampled pooled supragingival plaque in carefully controlled ALL and healthy populations, and analyzed its microbial composition via 16S-based 454 pyrosequencing. Oral health conditions, except for periodontal conditions, were well balanced between the ALL patients and healthy groups, further validating the use of supragingival plaque as a representative microbiota to delineate the influence of the immune status on oral microbiome.

The holistic pattern of leukemia pediatric patients was presented and compared with that of healthy controls, revealing key characteristics of oral microbiota associated with childhood ALL. ALL patients had lower richness and less diversity of oral microbiota compared with healthy subjects. Similar results were reported by Sixou et al, in which the microbial composition of supragingival plaque from the leukemia patients, analyzed by traditional culture methods, were found to be less complex than that of healthy controls [10]. However, Cargill et al. found that the complexity of oral microbiota was not statistically different between leukemia patients and healthy controls until initiation of antineoplastic treatment [33]. This inconsistency might be attributed to techniques used to profile the oral microbiota. Previous studies, mainly using culture methods, based their results on a few selected cultivable bacteria, neglecting characteristics of other bacteria in oral cavity of leukemia patients. At the time of sampling, oral bacteria might have already undergone selection after the disease manifested, with some bacteria inhibited and others thriving, resulting in divergent conclusions from different studies which focused on a few types of bacteria. By using 454 pyrosequencing, a holistic pattern of oral microbiome was presented and bacteria with low abundance and under the detection limit of culture methods could be investigated [36]. A decrease in intestinal microbial diversity was found in pediatric patients with acute myeloid leukemia [37]. The decrease of richness and diversity observed in the current study points to dysbiosis of oral microbiota in ALL patients. In addition to richness and diversity, we report altered structure and composition of oral microbiota in ALL patients. Altered structure and composition of oral microbiota in ALL patients were related to health status of investigated subjects. This was revealed in the separation pattern of samples based on cluster analyses, which were consistent with the presence/absence of leukemia. Since age, dentition stage, plaque pH, plaque index and caries status (dmfs/DMFS) were comparable between ALL patients and healthy children, microbial differences observed in supragingival plaque are likely directly caused by leukemia itself.

It is well-known that the host and microbiota have a dynamic interaction, which is closely associated with health [38]. The host immune system is believed to play an important role in shaping human microbiota [39]. A study on the bacterial and fungal microbiota of the stomach fluid indicated that immune status plays a major role in shaping the gastric fluid microbiota in terms of both diversity and composition [40]. Important effectors of innate immunity, α-defensins that are a kind of secreted antibacterial protein produced by epithelial cells, can affect the composition of intestinal microbial communities [41], [42]. Immune-driven dysbiosis in the intestine has been found in mice deficient for the transcription factor T-bet, which is associated with both the innate and the adaptive immune system [2]. Here we showed the importance of the immune system in shaping the oral microbial structure. This is not surprising, because like the small and large intestine, the lamina propria of oral mucosa typically has organized and diffuse lymphoid tissues [43]. In ALL patients, the disorder of lymphoid progenitor cells, the major part of the body's immune system, can lead to impairment of the host immunity [14], [15]. The malignant proliferation of lymphocytes in bone marrow infiltrates into the peripheral lymphoid tissues including the lymphoid tissue of oral mucosa, leading to impairment of oral immunity [7], [44]. Furthermore, the salivary defense system which contains various antimicrobial components such as secreted immunoglobulin A (SIgA), α-amylase and lysozyme, has been reported to be significantly impaired in leukemia [45]. However, the exact mechanisms involved in the interaction between the oral immune system and microbiota need further research.

Our investigations of oral microbiota in ALL patients also provided the opportunity for identifying potential microbiota associated with systemic infections in leukemia patients. The present data suggest two taxonomical lineages (*Firmicutes/Bacilli/Lactobacillales/Carnobacteriaceae/Granullicatella*, and *Firmicutes/Bacilli/Lactobacillales/Aerococcaceae/Abiotrophia*) that are much more abundant in the supragingival plaque of ALL patients than healthy controls from the phylum down to the genus level; this indicates that favorable conditions existed for their development in the oral cavity of ALL patients. This could be responsible for an increased risk of bacteremia in leukemia patients. *Granulicatella* and *Abiotrophia*, formerly known as nutritionally variant streptococcus (NVS), have been commonly implicated in endocarditis and bacteraemia, and also in several other infections such as central nervous system infections, otitis media, cholangitis and arthritis [46], [47]. A high mortality rate for endocarditis by NVS has been reported [48], [49]. Previous studies found that oral bacteria are responsible for 25% to 50% of systemic infections in neutropenic patients [50]. An increase in abundance of opportunistic pathogens might be an important factor to the increased risk of systemic infections in leukemia patients. Altered health status in ALL patients predisposes the host to developing oral infection by favoring pathogenic bacteria thriving in the oral niche. Prevention of oral microbiota dysbiosis might be a promising measure for decreasing mortality in patients with ALL. It should be noted that the 16S rRNA gene assay has its own limitation, i.e. the detection of 16S rRNA sequences does not imply that live bacteria are present. Therefore, either combination of 16S rRNA gene assay with traditional culture-based approach, or further cohort studies are needed to elucidate the exact relationship between oral microbial disequilibrium and risk of systemic infection in ALL-affected patient.

## Conclusion

In summary, by comparing the oral microbial composition of ALL patients and healthy subjects, we have identified a structural imbalance of the oral microbiota, characterized by reduced diversity of microbiota and abundance alterations of certain bacteria, possibly involved in systemic infections, indicating the important role immune status plays in shaping the structure of oral microbiota. Although more works still need to be done, the dysbiosis of oral microbiota identified in this study provides insight into the host-microbe interactions related to the infectious complications of this susceptible population.

## Supporting Information

Figure S1 **Heatmap analysis of the orders detected among all subjects based on microbiota composition and abundance.** The colour of each column represents relative abundance of the corresponding order according to the scale at the bottom of the plot. Subject metadata are shown using checkerboard plots based on the following color codes: ALL subjects (black) or healthy children (white); <6 years old (white), 6–12 years (gray) or older than 12 years (black); male (black) or female (white); primary (white), mixed (gray) or permanent (black) dentition; plaque index: <1.00 (white), 1.00–2.00 (gray) or >2.00 (black); plaque pH: <5.4 (white), 5.4–5.7 (gray) or >5.7 (black); dmfs/DMFS: 0–3 (white), 4–7 (gray) or 8–11 (black); gingivitis presence (gray) or absence (white). See figure 1 and table S1 for additional details. H, healthy children. L, acute lymphoblastic leukemia affected children.
(TIF)

Figure S2 **Relative abundance of oral microbiota compositions at the phylum level.** Comparison of all samples from ALL patients and healthy subjects showed major phyla comprised of *Proteobacteria*, *Firmicutes*, *Fusobacteria*, *Actinobacteria*, *Bacterioidetes* and candidate division TM7. H, healthy children, L, acute lymphoblastic leukemia-affected children.
(TIF)

Table S1 **Overview of subject clinical information.**
(DOC)

Table S2 **The number of OTUs and species richness and diversity estimates in each supragingival plaque microbiome.**
(DOC)

Table S3 **Differential relative abundance of bacterial taxonomy profiles of acute lymphoblastic leukemia (ALL) patients and healthy (H) subjects based on Metastats analysis.**
(DOC)
