# Correction: Oral Microbiota Distinguishes Acute Lymphoblastic Leukemia Pediatric Hosts from Healthy Populations

**DOI:** 10.1371/journal.pone.0110449

**Published:** 2014-10-03

**Authors:** The PLOS ONE Staff

The seventh author’s name is spelled incorrectly. The correct name is: Jinzhi He.
