# Supplementary figures and images for: Oral Microbiota Distinguishes Acute Lymphoblastic Leukemia Pediatric Hosts from Healthy Populations
Source: PLoS One. 2014 Jul 15;9(7):e102116. doi: 10.1371/journal.pone.0102116 (PMC4099009; doi:10.1371/journal.pone.0102116)

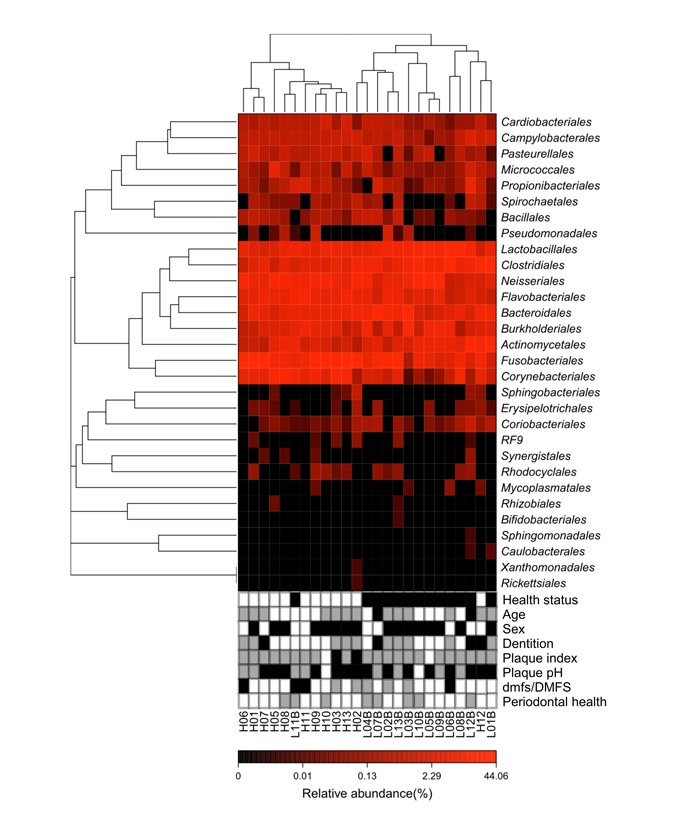

Supplement: Figure S1 — Heatmap analysis of the orders detected among all subjects based on microbiota composition and abundance. The colour of each column represents relative abundance of the corresponding order according to the scale at the bottom of the plot. Subject metadata are shown using checkerboard plots based on the following color codes: ALL subjects (black) or healthy children (white); <6 years old (white), 6–12 years (gray) or older than 12 years (black); male (black) or female (white); primary (white), mixed (gray) or permanent (black) dentition; plaque index: <1.00 (white), 1.00–2.00 (gray) or >2.00 (black); plaque pH: <5.4 (white), 5.4–5.7 (gray) or >5.7 (black); dmfs/DMFS: 0–3 (white), 4–7 (gray) or 8–11 (black); gingivitis presence (gray) or absence (white). See figure 1 and table S1 for additional details. H, healthy children. L, acute lymphoblastic leukemia affected children. (TIF) [file pone.0102116.s001.tif]

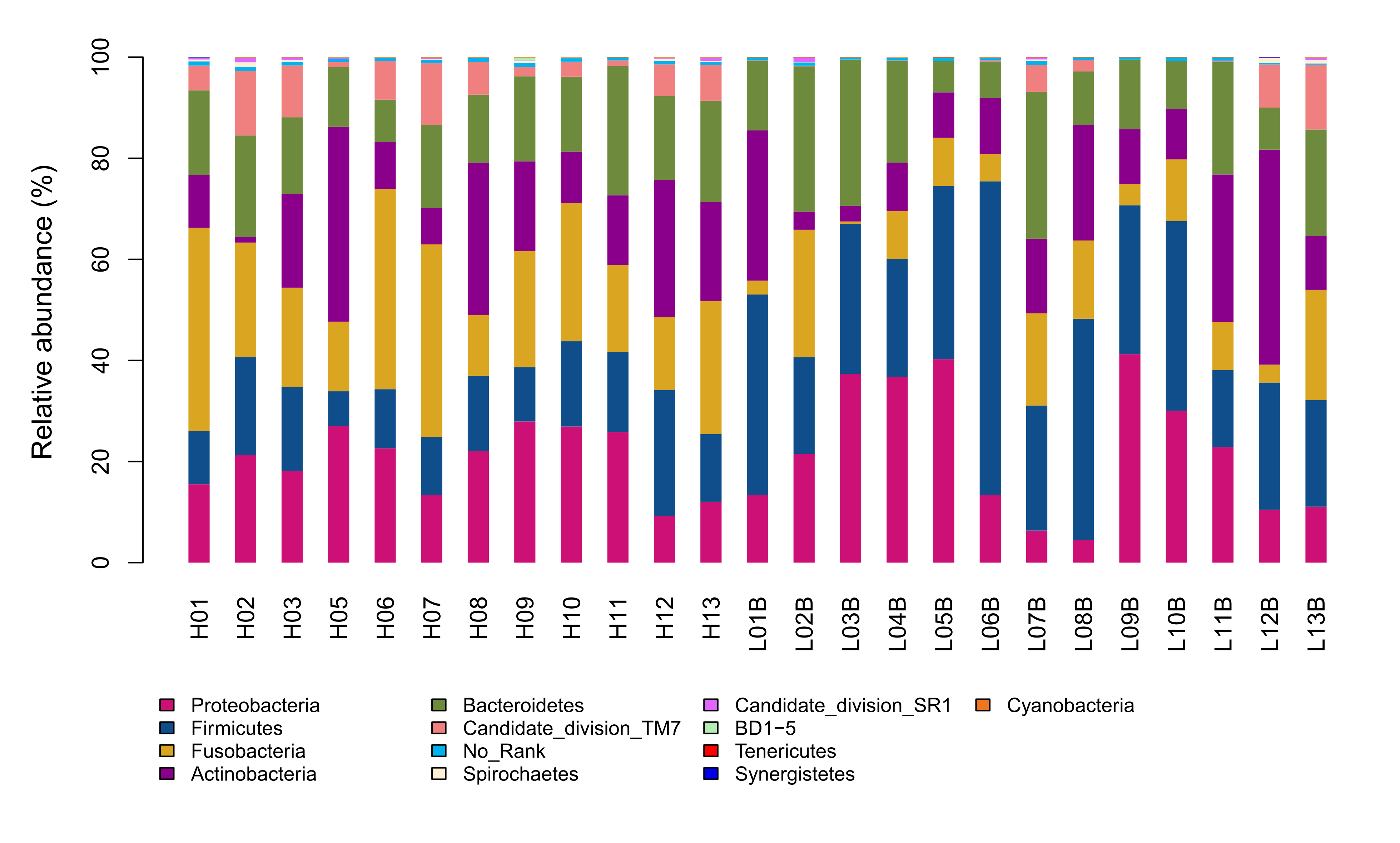

Supplement: Figure S2 — Relative abundance of oral microbiota compositions at the phylum level. Comparison of all samples from ALL patients and healthy subjects showed major phyla comprised of Proteobacteria, Firmicutes, Fusobacteria, Actinobacteria, Bacterioidetes and candidate division TM7. H, healthy children, L, acute lymphoblastic leukemia-affected children. (TIF) [file pone.0102116.s002.tif]
