# Supplementary material for: Oral Microbiota Distinguishes Acute Lymphoblastic Leukemia Pediatric Hosts from Healthy Populations
Source: PLoS One. 2014 Jul 15;9(7):e102116. doi: 10.1371/journal.pone.0102116 (PMC4099009; doi:10.1371/journal.pone.0102116)
Supplement: Table S1 — Overview of subject clinical information. (DOC) [file pone.0102116.s003.doc]

**Table S1. Overview of subject clinical information. dmfs/DMFS, Decayed-Missing-Filled Surfaces Index in primary and permanent teeth, respectively. F, female. G, gingivitis. H, healthy children, L, acute lymphoblastic leukemia affected children. M, male. ND, no data obtained.**

| **Sample ID** | **Age** | **Gender** | **Dentition** | **Plaque index** | **Plaque pH** | **dmfs/DMFS** | **Periodontal condition** | **ALL subtypes based on immunophenotypes** | **Systemic antibiotics within 3 months** | **Local antimicrobial treatment within 2 weeks** |
| --- | --- | --- | --- | --- | --- | --- | --- | --- | --- | --- |
| H01 | 11 | M | Mixed | 1.63 | 5.7 | 1 | - | - | No | No |
| H02 | 7 | M | Mixed | 2.92 | 5.8 | 5 | - | - | No | No |
| H03 | 7 | M | Mixed | 2.92 | 6.0 | 5 | - | - | No | No |
| H04 | 5 | F | Primary | 1.63 | 5.8 | 3 | - | - | No | No |
| H05 | 5 | M | Primary | 1.13 | 6.0 | 2 | - | - | No | No |
| H06 | 6 | F | Mixed | 1.13 | 5.7 | 10 | - | - | No | No |
| H07 | 12 | F | Permanent | 1.25 | 6.1 | 3 | - | - | No | No |
| H08 | 5 | M | Primary | 1.16 | 5.8 | 0 | G | - | No | No |
| H09 | 3 | M | Primary | 1.25 | 6.0 | 2 | - | - | No | No |
| H10 | 6 | M | Primary | 0.75 | 5.3 | 0 | G | - | No | No |
| H11 | 4 | F | Primary | 1.25 | 5.7 | 8 | - | - | No | No |
| H12 | 12 | F | Permanent | 1.25 | 6.0 | 0 | - | - | No | No |
| H13 | 6 | M | Mixed | 1.13 | 6.1 | 0 | - | - | No | No |
| L01 | 9 | M | Mixed | 2.00 | 6.1 | 2 | - | Early pre-B cell | No | No |
| L02 | 8 | M | Mixed | 1.63 | 5.9 | 5 | - | B cell | No | No |
| L03 | 8 | M | Mixed | 1.50 | 5.9 | 5 | G | ND | No | No |
| L04 | 5 | F | Primary | 2.00 | 5.9 | 4 | G | Pre-B cell | No | No |
| L05 | 4 | M | Primary | 0.75 | 6.0 | 3 | - | B cell | No | No |
| L06 | 8 | F | Mixed | 2.00 | 5.8 | 11 | - | B cell | No | No |
| L07 | 13 | F | Permanent | 1.29 | 5.7 | 3 | G | Pre-B cell | No | No |
| L08 | 2 | M | Primary | 1.50 | 5.6 | 0 | - | Early pre-B cell | No | No |
| L09 | 2 | M | Primary | 1.17 | 5.7 | 3 | - | Pre-B cell | No | No |
| L10 | 5 | M | Primary | 1.08 | 5.1 | 0 | G | Early pre-B cell | No | No |
| L11 | 3 | F | Primary | 1.33 | 5.6 | 8 | G | Early pre-B cell | No | No |
| L12 | 14 | F | Permanent | 1.00 | 6.1 | 0 | G | Early Pre-B cell | No | No |
| L13 | 6 | M | Mixed | 1.04 | 5.4 | 1 | - | B cell | No | No |
