# Supplementary material for: Oral Microbiota Distinguishes Acute Lymphoblastic Leukemia Pediatric Hosts from Healthy Populations
Source: PLoS One. 2014 Jul 15;9(7):e102116. doi: 10.1371/journal.pone.0102116 (PMC4099009; doi:10.1371/journal.pone.0102116)
Supplement: Table S2 — The number of OTUs and species richness and diversity estimates in each supragingival plaque microbiome. (DOC) [file pone.0102116.s004.doc]

**Table S2. The number of OTUs and species richness and diversity estimates in each supragingival plaque microbiome.**

| **Sample ID** | **ACE** | **Chao 1** | **Simpson** | **Reads** | **OTUs (0.03 dissimilarity level)** |
| --- | --- | --- | --- | --- | --- |
| H01 | 16434.05 | 9583.64 | 0.0151 | 23773 | 3835 |
| H02 | 15501.17 | 9172.62 | 0.0186 | 23489 | 3820 |
| H03 | 14531.74 | 8843.23 | 0.0186 | 23476 | 3460 |
| H05 | 13628.65 | 8038.94 | 0.0194 | 25928 | 3315 |
| H06 | 10347.32 | 5703.39 | 0.0398 | 15126 | 2218 |
| H07 | 13353.94 | 7737.81 | 0.0187 | 16933 | 2878 |
| H08 | 12602.89 | 6813.38 | 0.0112 | 13000 | 2423 |
| H09 | 12223.16 | 6733.17 | 0.0095 | 12697 | 2481 |
| H10 | 12383.07 | 7446.54 | 0.0108 | 16070 | 2910 |
| H11 | 9252.03 | 5420.97 | 0.0137 | 12553 | 2227 |
| H12 | 10227.73 | 5954.40 | 0.0172 | 13029 | 2302 |
| H13 | 8984.14 | 5133.22 | 0.0095 | 10079 | 2013 |
| L01B | 6145.18 | 3835.86 | 0.0593 | 11790 | 1572 |
| L02B | 9052.79 | 4714.46 | 0.0246 | 8680 | 1611 |
| L03B | 5805.36 | 3645.80 | 0.0741 | 10872 | 1398 |
| L04B | 9439.94 | 4907.17 | 0.0390 | 9395 | 1714 |
| L05B | 7611.93 | 4319.02 | 0.0607 | 10578 | 1541 |
| L06B | 5176.04 | 3117.42 | 0.0384 | 6234 | 1187 |
| L07B | 7447.02 | 3654.66 | 0.0146 | 5734 | 1412 |
| L08B | 6418.03 | 3432.33 | 0.0175 | 4801 | 1156 |
| L09B | 5109.52 | 2724.07 | 0.0792 | 5600 | 816 |
| L10B | 5180.77 | 2924.10 | 0.0606 | 5873 | 1026 |
| L11B | 14356.19 | 8480.24 | 0.0160 | 24881 | 3467 |
| L12B | 11810.32 | 7196.95 | 0.0152 | 22770 | 2821 |
| L13B | 14418.47 | 8645.49 | 0.0120 | 22530 | 3389 |
