# Supplementary material for: Oral Microbiota Distinguishes Acute Lymphoblastic Leukemia Pediatric Hosts from Healthy Populations
Source: PLoS One. 2014 Jul 15;9(7):e102116. doi: 10.1371/journal.pone.0102116 (PMC4099009; doi:10.1371/journal.pone.0102116)
Supplement: Table S3 — Differential relative abundance of bacterial taxonomy profiles of acute lymphoblastic leukemia (ALL) patients and healthy (H) subjects based on Metastats analysis. (DOC) [file pone.0102116.s005.doc]

**Table S3. Differential relative abundance of bacterial taxonomy profiles of acute lymphoblastic leukemia (ALL) patients and healthy (H) subjects based on Metastats analysis. Taxa shown in red had significantly higher relative abundance in ALL patients compared with healthy subjects, taxa shown in blue had significantly lower abundance and those without significant differences are shown in black. Only taxonomic lineages containing at least one taxa of significant difference in relative abundance between the two groups are presented. Taxa with relative abundance < %1 in both groups are not shown. (*p* value threshold = 0.05; q value threshold = 0.5)**

| **Phylum** | **Class** | **Order** | **Family** | **Genus** |
| --- | --- | --- | --- | --- |
| Firmicutes | Bacilli | Lactobacillales | Carnobacteriaceae | Granullicatella |
|  |  |  | Aerococcaceae | Abiotrophia |
|  | Negativicutes | Selenomonadales | Veillonellaceae | Veillonella |
| [Fusobacteria](http://www.ncbi.nlm.nih.gov/Taxonomy/Browser/wwwtax.cgi?mode=Undef&id=32066&lvl=3&keep=1&srchmode=1&unlock) | [Fusobacteriia](http://www.ncbi.nlm.nih.gov/Taxonomy/Browser/wwwtax.cgi?mode=Undef&id=203490&lvl=3&lin=f&keep=1&srchmode=1&unlock) | [Fusobacteriales](http://www.ncbi.nlm.nih.gov/Taxonomy/Browser/wwwtax.cgi?mode=Undef&id=203491&lvl=3&lin=f&keep=1&srchmode=1&unlock) | Fusobacteriaceae |  |
|  |  |  | [Leptotrichiaceae](http://www.ncbi.nlm.nih.gov/Taxonomy/Browser/wwwtax.cgi?mode=Undef&id=1129771&lvl=3&lin=f&keep=1&srchmode=1&unlock) | Leptotrichia |
| Proteobacteria | Betaproteobacteria | Burkholderiales | [Comamonadaceae](http://www.ncbi.nlm.nih.gov/Taxonomy/Browser/wwwtax.cgi?mode=Undef&id=80864&lvl=3&lin=f&keep=1&srchmode=1&unlock) | Comamonas |
